# Intensified inequities: Young people's experiences of Covid‐19 and school closures in Uganda

**DOI:** 10.1111/chso.12627

**Published:** 2022-09-05

**Authors:** Simone Datzberger, Jenny Parkes, Amiya Bhatia, Rehema Nagawa, Joan Ritar Kasidi, Brian Junior Musenze, Dipak Naker, Karen Devries

**Affiliations:** ^1^ UCL Institute of Education London UK; ^2^ London School of Hygiene & Tropical Medicine (LSHTM) London UK; ^3^ Medical Research Council/Uganda Virus Research Institute Entebbe Uganda; ^4^ Makerere Institute of Social Research Makerere University Kampala Uganda; ^5^ Raising Voices Kampala Uganda

**Keywords:** Covid‐19, education, inequities, intersectionality, Uganda, young people

## Abstract

Uganda had the longest period of school closures worldwide as a response measure during the Covid‐19 pandemic. Drawing on longitudinal qualitative data from the Contexts of Violence in Adolescence Cohort Study (CoVAC) (2018–2023), we examine how this has affected the lives of adolescents in Uganda. Our analysis showcases how intersecting inequities based on socioeconomic circumstances, gender and location have intensified, with detrimental effects on young people's educational paths and life circumstances. Strategies that take the intersections of these inequities into account are urgently needed to help the most disadvantaged and marginalized young people return to school.

## INTRODUCTION

Studies on the predicted and already visible impact of Covid‐19 on education in SSA (Sub‐Saharan Africa) reveal how inequities that affected children and their families prior to the pandemic have intensified during and after school closures (e.g. Alam & Tiwari, 2021; de Klerk & Palmer, 2021; Edwards, 2021; Save the Children International, 2021; UNICEF, 2021). This work has provided us with important insights on the many challenges in children's and young people's lives resulting from school closures. The majority of research^1^ on the effects of Covid‐19 in education in Africa has been conducted in South Africa (e.g. Duby et al., 2022; Landa et al., 2021), with a strong emphasis on learning technologies (e.g. Mhlanga & Moloi, 2020) or teachers' experiences (e.g. Williams et al., 2022). Less attention has been paid to how young people's specific circumstances in low‐income contexts were associated with different experiences of the pandemic. We, therefore, analyse how the effects of the pandemic, in particular the prolonged closure of schools, have been experienced in varying ways by young people in Uganda, influenced by intersecting *socioeconomic circumstances*, *gender* and *location*. We illustrate why an intersectional lens is useful to recognize the diverse needs and very specific, often complex, vulnerabilities of children and youth in extreme situations, such as the Covid‐19 pandemic. We further showcase why an intersectional lens has the potential to inform more tailored approaches to help young people cope with the loss of schooling. Our case study is Uganda, which has enforced one of the longest period of school closures (83 weeks) due to Covid‐19.^2^ Our guiding question is: *How have school closures during Covid‐19 affected the life trajectories of young people in Uganda*?

We draw on the experiences of 22 research participants (10 males, 12 females), predominantly from a low socioeconomic status, who were either in school, planned to re‐enrol in school or in vocational training after the first lockdown. One of our in‐depth case studies relates to a female participant we call Atala (pseudonym). Her circumstances prior and during the pandemic serve as an example of a particularly marginalized and disadvantaged young female, affected by multiple and intersecting conditions. Our study participants were aged between 14 and 18 years when we first met them in 2018, and were 17–21 years old when we last spoke to them in 2021. Over the course of the past 4 years, we interviewed each participant six times (see Annex 2). The transition from early to late adolescence (or early adulthood) is a crucial time for young people, characterized by significant physical, psychological and social changes (WHO, 2020). For the young people in our study, this critical period of their lives has been interrupted by Covid‐19 lockdowns, with severe effects on their educational paths, well‐being, socioeconomic circumstances and life trajectories.

In the next section of this paper, we delineate our theoretical framework, explaining why we apply an intersectional lens to better assess and understand how inequities have intensified for youth because of school closures. We describe our research methods and provide more background of the broader research project. Before discussing the young people's individual accounts, we look into already existing inequities and challenges in education before Covid‐19 in Uganda to better understand how these not only intensified but also led to varied experiences during the pandemic. We then turn to our study participants to analyse their perspectives on how school closures have affected their life paths.

## INEQUITY FROM AN INTERSECTIONAL LENS

Theoretically we are interested in how the prolonged closure of schools during the pandemic in Uganda has served to entrench different inequities for marginalized youth, prompted by the intersection of specific conditions. As we explain in the subsequent section, centring our analysis on inequity allows us to focus on social injustices that arose or were aggravated during the pandemic, while at the same time reflecting on the conditions and necessities young people feel are needed to safely return to school. Analysing these social injustices and experiences of marginalized youth from an intersectional lens, helps us to trace interconnections and interdependencies between social categories and its surrounding system (Atewologun, 2018) to explore how these affect the material conditions and lived experiences of our participants.

### Conceptualizing inequity

**FIGURE 1:**
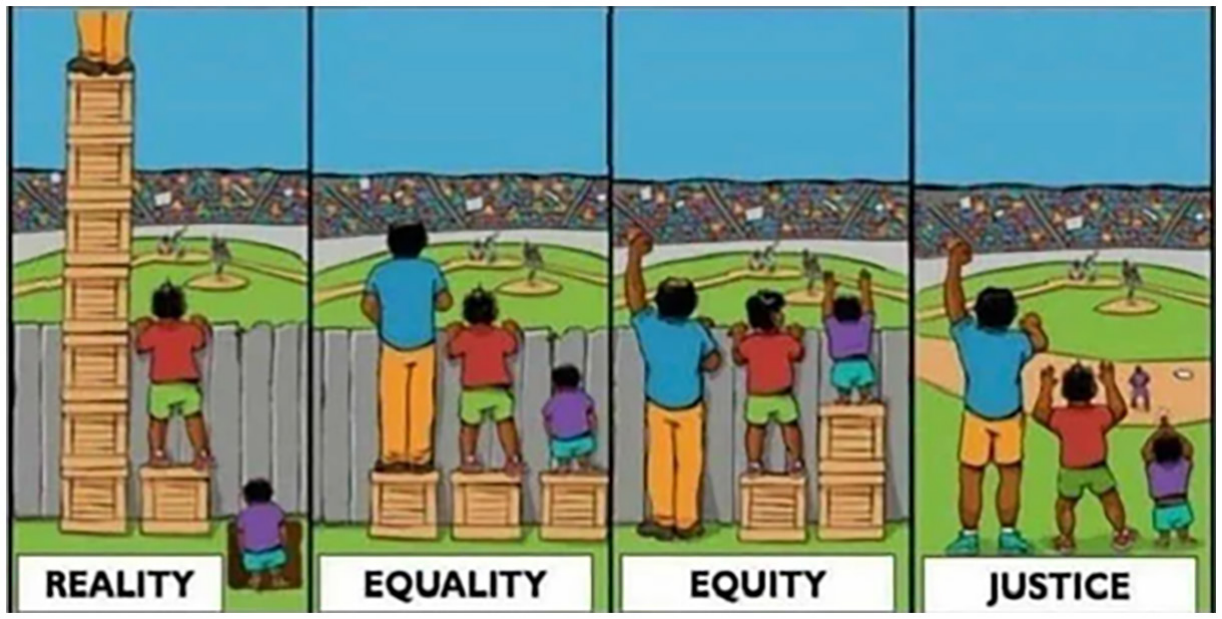
Source: https://nce.aasa.org/equity‐then‐and‐now/, accessed 02 May 2022

The concepts ‘inequity’ and ‘inequality’ or ‘equity’ and ‘equality’ are often used interchangeably. These terms are not synonymous, however (Bamberger & Segone, 2011; Braveman & Gruskin, 2003; Stewart, 2013). Equality generally refers to processes in which an individual or a group of people are given the same resources or opportunities. This could be for instance, free access to education and the provision of school books for all boys and girls. Equity‐based approaches, on the other hand, further take into account whether (or not) these processes are just and fair, considering, and responding to, children's specific circumstances which may challenge them from accessing free education in the first place, such as: disability, location (e.g. remote areas with no access to schools), or not having financial or moral support from a caregiver in their learning, etc. In other words, a specific focus on inequity recognizes that each person has and is surrounded by different (pre‐)conditions which affect the outcome of an intervention or policy. The aim of equity‐based approaches is to identify not just the same but also additional (or unique) resources and opportunities needed to reach a socially just and fair outcome (Bamberger & Segone, 2011, pp. 3–4). Hence, equity takes into account underlying social disadvantage (Braveman & Gruskin, 2003) and demands a consideration of how to change the structures that created this disadvantage, as shown in Figure 1.

Social, political, economic or cultural injustices, or disadvantages occur at both the individual and societal level. It is precisely here where an intersectional lens allows us to analyse, assess and shed light on differences in lived experiences among marginalized youth while also paying attention to the structures that young people live and learn in. It helps us to understand how multiple aspects causing disadvantage traverse, thereby challenging assumptions of within‐group homogeneity (Atewologun, 2018).

### Borrowing ‘intersectionality’ from black feminist thought

**FIGURE 2:**
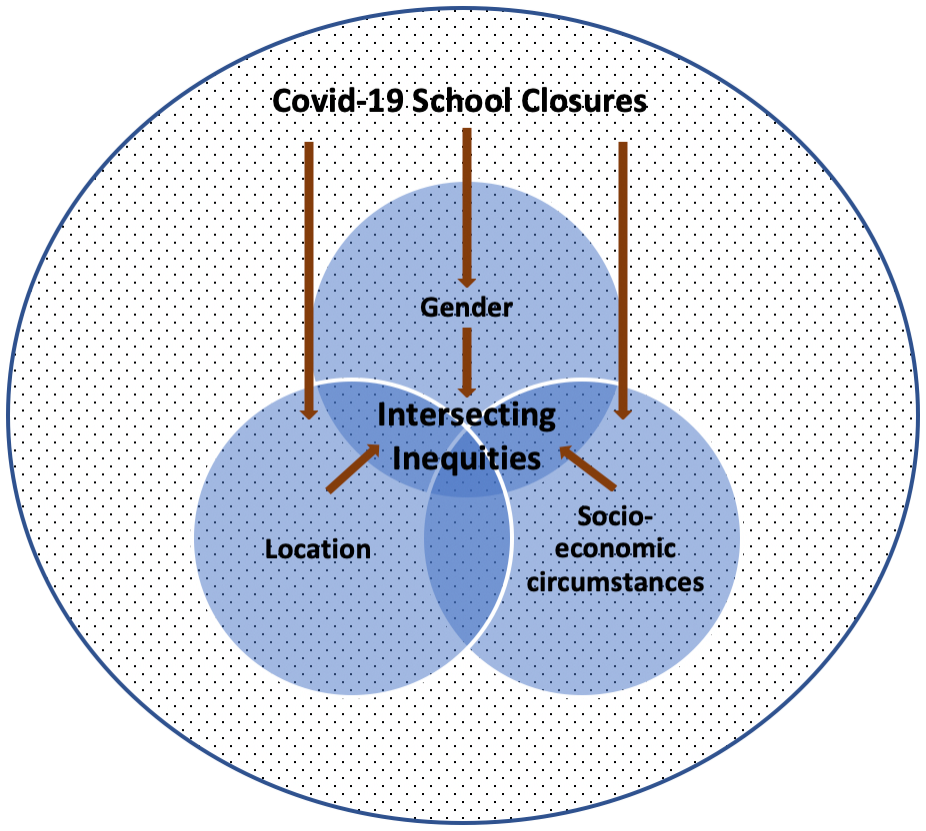
Intersecting inequities due to Covid‐19 school closures

Intersectionality as a qualitative analytical framework and a critical theory (Davis, 2008), has developed from the work of black feminists, including Kimberlé Williams Crenshaw (1989, 1991), who critiqued traditional feminist thought for ignoring the different experiences of black women grounded in racism and other forms of discrimination, especially if compared to white (often more privileged) females from the West. Since then, intersectionality has become one of the most noteworthy contributions of women's studies (Davis, 2008; McCall, 2005), by looking into the interaction between gender, race and other categories of difference in individual lives, social practices, institutional arrangements and cultural ideologies and the outcomes of these interactions in terms of power (Davis, 2008, p. 68). Intersectional enquiry is a generative tool for understanding contextual dynamics of power (Cho et al., 2013, p. 788). We chose an intersectional lens as we found it most useful to unravel the complexities within and interrelationships between inequities due to school closures experienced by marginalized youth in Uganda. Intersectionality helps us to analyse how categories of disadvantage are layered and interwoven, rather than considering each category on its own. It further helps us to avoid an essentialist stance, by paying attention to individual lived experiences and insights, as opposed to generalizing on the basis of just one category (e.g. ‘all’ girls or boys, or ‘all’ rural or urban youth), which often leads to one‐size fits all approaches ignoring young people's specific conditions, circumstances and needs. It also helps us to unravel how youth from low‐income contexts, are all of a sudden confronted with new and additional grievances and forms of marginalization they did not experience before the closure of schools. We focus in particular on the intersecting categories of ‘gender’, ‘location’ and ‘socioeconomic background’, which emerged as most significant in our preliminary data analysis. At the same time we recognize that categories are not distinct, but always permeated by other categories, and the dynamics of power (Cho et al., 2013, p. 795). As shown in Figure 2, we thus see the interplay of intersecting dimensions and intensified inequities as fluid and not static, as categories are context‐specific allowing for different adaptations of an intersectional lens. In fact, it is exactly this open‐endedness inherent in intersectional theory, that made it a (black feminist) success story in the first place (Davis, 2008).

## RESEARCH METHODS: CAPTURING THE VOICES OF MARGINALIZED ADOLESCENTS

Our study was conducted as part of a broader research project: Contexts of Violence in Adolescence Cohort Study (CoVAC). CoVAC is a mixed methodology cohort study that aims to build understanding on how family, peer, school and community contexts affect young people's experiences of violence in adolescence and early adulthood. It includes epidemiological data collection at three time points, and a qualitative longitudinal component, with fieldwork for 2–3 months each year from 2018 to 2022 in the Luwero District of Uganda. 36 young people (see Annex 1), now mainly aged 17–22 years, are the core participants in the qualitative component. The qualitative core participants were purposely selected from the project's quantitative cohort of 3431 young people, based on their responses in our wave 1 epidemiological survey in 2014 and prior agreement to be contacted again. The qualitative sample includes equal numbers of girls and boys, from rural and urban communities, and with varying experiences of violence (more or less severe) in their lives up that point (in 2014). Each core participant was assigned a ‘key’ researcher, who is Ugandan, engages with them exclusively in the local language (Luganda), and where possible same sex (and always same sex for female participants), enabling good research relationships to be sustained over time. Three of our study participants were not available for one round of data collection each (due to imprisonment or other challenging personal circumstances). We managed to still stay in touch with them, and maintain un‐interrupted contact with 33 of our cohort via regular stay‐in‐touch calls in‐between data collection. Carefully nurturing relationships of trust between the researchers and study participants has helped us to work with the same cohort of young people over the past 4 years.

**FIGURE 3:**
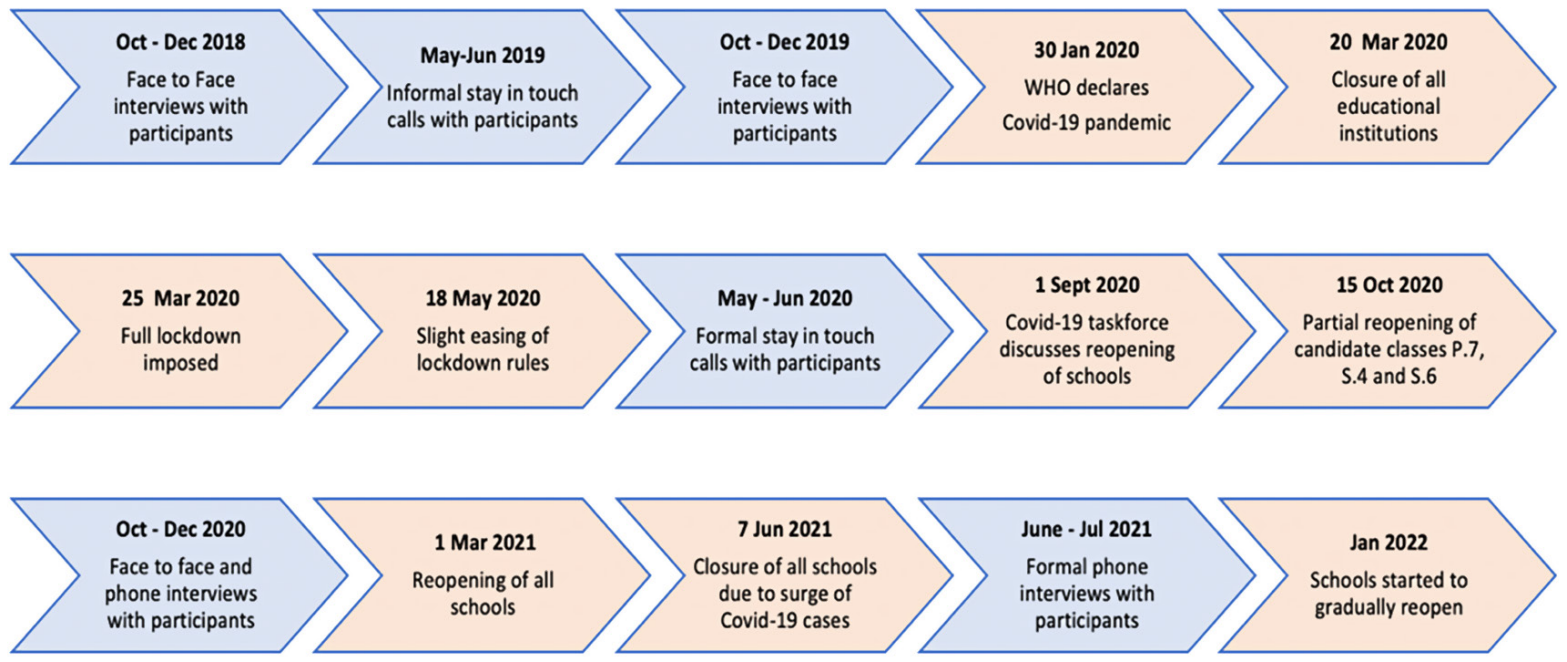
Timeline of interviews, Covid‐19 pandemic and school closures

Our qualitative data stem from six rounds of interviews and other encounters with the core participants since 2018 (see Annex 2). Prior to the pandemic we also conducted 10 FGDs (Focus Group Discussions) with our study participants and their peers. These FGDs allowed us to gain deeper insights into how surrounding structures, norms, institutions and interactions influence young people's capacity over time to resist the harmful effects of violence (in and outside their schooling experience). In addition, we met with and interviewed their caregivers (i.e. biological parents, extended family or close community members who look after them) and/or teachers. For this paper, we make use of data from all rounds of data collection, though we present and discuss predominantly findings from mobile phone interviews with the core participants, conducted May–June 2020, face‐to‐face fieldwork three interviews conducted October–December 2020, and phone interviews conducted May–August 2021. During our rounds of research since 2020, topics included the effects of Covid‐19 on their daily lives, on relationships in families, with friends, on schooling/work, and in communities, along with discussion about their coping strategies, sources of support and views on lockdown measures (see Figure 3 below).

For this paper, we draw on data from 22 (12 female, 10 male) study participants, who reported either being back in school, were planning to re‐enrol after the prolonged closure of schools, or decided to enrol in vocational training institutes (see Annex 1). The remaining 14 participants were already out of school prior to the first lockdown.

Data were translated (from Luganda to English) and transcribed by the research team, and then coded using NVivo. In view of the many challenging circumstances our participants faced prior and during the pandemic, all participants were offered counselling services. Whenever we identified severe forms of violence or emotional distress among our research participants, their close family members or peers, we followed an immediate referral process and offered counselling. Our study followed a strict ethics protocol (see Devries et al., 2020). During lockdowns, additional measures were adopted to address safety issues relating to remote data collection (via mobile phones). Potential risks were mitigated through regular trainings of researchers, and by adapting CoVAC's existing safety and referral plan to include provision of telephone counselling support. Researchers sought participants' views on preferred times and locations to speak and asked the participants to alert them if they needed to interrupt the discussions, being careful to also listen out for any signs of distress or discomfort. The researchers also took extra care and caution around probing, asking open‐ended questions and avoiding direct questions on personal experiences of violence so that the participants were able to maintain control over any personal disclosures.

### Participants' ‘voices’

While centring our analysis on young people's accounts as a research method can be emancipatory, we acknowledge that the researcher often benefits more from the telling than the researched (Kincheloe & Mclaren, 2011). To counter this trend, our research methods and approach share some similarities with participatory action research (PAR) methods (e.g. Halliday et al., 2019), in that we aim to democratize knowledge by focusing on young people's perceptions and viewpoints as our main unit of analysis. However, data and findings do not translate into immediate educational interventions or new educational programming co‐led or co‐developed by youth, as would be the case in traditional PAR projects. Rather, as a research collaboration between research organizations and a local Non‐governmental Organization (NGO), Raising Voices,^3^ we are interested in feeding back insights into public discourse and informing policy decisions.

Our analysis developed through regular team discussions on emerging findings alongside analysis of the coded data by thematic areas. The composition of our research team consisting of Ugandan and non‐Ugandan researchers helped to combine outsider with insider perspectives and contemplating findings from various angles and perspectives as a team. In addition, and central to our analysis, we read the interview transcript for each young person in conjunction with the biographical narrative data already collected, enabling us to look closely at how Covid‐19 in combination with prolonged closures of schools has affected the life trajectories of the young people we are in conversation with.

## UGANDA'S EDUCATION SECTOR PRIOR TO COVID‐19

Uganda inherited its current education system from its former British colonial administration. The appropriation of western and former colonial education systems by SSA countries have been questioned and critiqued by many—in particular, African scholars (e.g. Assié‐Lumumba, 2016; Kanu, 2003; Ngũgĩ wa Thiong'o, 1992). Uganda's significant investments and policy reforms since 1997 in education have not translated into the expected results with regards to poverty reduction through human capital investment (Datzberger, 2018). For instance, school drop‐out rates in Uganda were already extremely high prior to the pandemic, with 64.5% of primary school children dropping out before reaching the last grade of primary education.^4^ Dropping out of school is generally associated with a range of intersecting aspects rather than being caused by one single event. These include: socioeconomic circumstances (economic well‐being, education of the household head, place of residence), child labour, poverty, lack of support from parents, death, pregnancy, fosterage, poor performance in school, lack of support from teachers, sickness or disability (Ampiah & Adu‐Yeboah, 2009; Kakuba et al., 2021; World Bank, 2017). All of this is accompanied by the many (hidden) costs for schooling. Although Uganda has implemented UPE (Universal Primary Education) and USE (Universal Secondary Education) policies, education is not free (Kakuba et al., 2021; Omoeva & Gale, 2016) and many structural barriers in accessing formal education persist (Datzberger, 2018, 2022). Prior to the pandemic, in 2017, Uganda's Gross Enrolment Rate^5^ in secondary education was 41%, and higher for males (43%) than females (38%) (UBOS, 2017, p. 29). Males were not only more likely to transition from primary to secondary schooling, but also to complete S.4 (Senior)^6^ as well as transition to S.5 compared to their female counterparts (UBOS, 2020, p. 26). The quality of education at both primary and secondary level is not only extremely low but has not improved over time (see Uwezo, 2019 for more details). At the same time, programs specifically designed to reduce high youth unemployment rates have, thus far, failed to respond to the many educational needs of youth and the local economy (Datzberger, 2018). In a study, which surveyed 500 Ugandan youth in 2016 about their educational experience in four different regions of the country, education was not perceived as having the same empowering effects for everyone when it comes to skills for employment or income‐generating activities (Datzberger, 2022). Respondents critiqued their schooling as being too theory‐focused and lacking practical or utilitarian aspects to overcome the many economic challenges they face (ibid.). Overall, formal education is often not responsive to the actual economic conditions, cultural context and political environment young people are facing (Datzberger, 2018, 2022; Datzberger & Parkes, 2021).

## ON THE EFFECTS OF SCHOOL CLOSURES ON YOUNG PEOPLE'S LIVES

The Covid‐19 pandemic has put multiple additional challenges in young people's lives with schools first closed in late March 2020 followed by partial reopening for candidates and finalists (P.7, S.4 and S.6) as of mid‐October 2020. For the majority of students who were not sitting national exams (more than 90%), schools remained closed for almost a year during the first lockdown. When schools finally started to re‐open in a staggered manner between March and June 2021,^7^ Uganda faced a surge of Covid‐19 cases and all educational institutions were forced to close again (Reuters, 2021). The country eventually enforced the longest school‐closure worldwide,^8^ with schools either fully or partially closed for 83 weeks (22 months) and starting to gradually reopen in January 2022.

While the long‐term effects of the pandemic can only be speculated, the immediate effects on children and youth have been devastating. Recent statistical models predict a learning deficit of 2.8 years in Uganda (Angrist et al., 2021). According to the UBOS (Ugandan Bureau of Statistics) there has been an increase of child labour from 21% to 36%, affecting in particular girls, with an increase from 20% before to 37% during Covid‐19, compared to boys with an increase from 22% before to 35% during the pandemic (UBOS, 2021 p. 43 and p. 86; see also Human Rights Watch, 2021). As far as secondary school net enrolment is concerned (i.e. percentage of adolescent youth of the official age group for a given level of education enrolled in secondary school), recent data points to a substantial drop in enrolment (UBOS, 2021). There have also been widespread reports of increases in teenage pregnancies since school lockdowns (UNPFA Uganda, 2021).

All of our school‐going adolescents experienced significant disruption in their education as a consequence of the pandemic. During the first lockdown they worried about their caregivers' capacity to send them back to school, due to the significant loss of income most of them faced. Some were concerned about having to repeat the school year or failing their exams.

I was not happy because I had just paid my full school fees and that does not get refunded to you (…) and I could not ask them [the school] to refund my fees. I was not happy because we were thrown back, there are these 2 months that we really read our books thinking that they would ask us to get back to school anytime, but when we saw that we were in the 3rd month of lockdown we gave up. (Kato, male, age 18).

It has affected me so much because our schooling has stopped, we no longer study and our parents no longer work, we do not get the necessities as much as we used to get them before. (Nkola, female, age 18).

I would be in school, but see I am now in the village. I came here with my mother because life was being a little difficult in town, then my mother decided that we go to the village where we could get free food. Our business was put at a standstill yet we needed to feed every day. (Ruth, female, age 18).

When we met again in Autumn 2020, those participants in candidate classes (S.4 and S.6) were able to re‐join school for a period, but their insecurities and anxieties suddenly re‐emerged when the second lockdown and renewed closure of schools was announced in early June 2021. Some, like Cathy, had just returned to school, shortly before the second closure was announced. She told us that if school closures go on for longer than 42 days (which later turned out to be the case), she will drop out, notwithstanding her initial aspirations to complete her A‐Levels.

Int.: God forbid, but what if this lockdown is extended for more 6 months, what…

Cathy: Shaaaaaaa, I just enrol for a course (…) Hmmmm what is that called? You revise as you forget what you revised, then you revise again as you forget, Aaaah that then becomes meaningless. Trust me if this happened then I will drop out.

Among our female study participants, not just financial constraints but also age seemed to play a role with young women feeling they were too old to re‐enter schooling following prolonged closure of schools. This may potentially further widen the gender gap in secondary school completion rates in the shorter and longer term. Nyanja, a male aged 19, also worried about his prospects for continuing with his education. He is among the very few study participants whose socioeconomic status could not be initially categorized as ‘poor’ or living in ‘extreme (multidimensional) poverty’ (see Alkire & Santos, 2013) prior to the pandemic. He had managed to resume his education after the first lockdown. However, when we spoke to him during the second closure of schools in early summer 2021, Nyanja's situation significantly worsened. He expressed great concerns about his caregivers' ability and willingness to further support his education, with two lockdowns having taken their toll on his family's socioeconomic standing. Nyanja's father, a farmer, who could take care of his family well prior to Covid‐19, now struggled in making an income. In Autumn 2020, when schools for candidate classes re‐opened, Nyanja told us that his father failed to pay for his fees. After the second school closure, Nyanja told us that he will not return to school, pointing again to the significant loss of his father's income. Over the years, we got to know Nyanja as a bright student, whose dream to become a doctor (to follow his grandfather's steps) did seem to be a realistic aspiration. Nyanja currently engages in casual work hoping to enrol in a vocational institute in the future, if at all.

While Nyanja's and Cathy's experiences are typical of the effects of Covid‐19 among study participants, some were even more deeply affected by Covid‐19 measures and regulations. In the subsequent sections, we apply our theoretical framework to examine how the prolonged closure of schools has led to varying intersecting inequities. We provide an in‐depth cases study of one participant, Atala, as her experience illustrates how the intersections of being female, living in a remote area and facing difficult socioeconomic circumstances—all of which already affected her life's trajectory prior to the pandemic—considerably aggravated her situation in the scope of two lockdowns, prolonged closure of schools and the pandemic. This will be followed by a discussion on how the combination of socioeconomic background and location challenged adolescents in accessing distance learning materials. Finally, we elaborate on how the intersection of gender and socioeconomic circumstances deepened pre‐existing inequities during school closures.

### On the intersections of socioeconomic circumstances, gender and location during school closures. Atala's experience

Atala (aged 19) is the oldest of five children, raised by and living with her mother, who sells tea in the streets of Kampala to make ends meet. Her father left the family when she was a small child, and has not provided any support since then, though Atala reported that his occasional visits have resulted in her mother's further pregnancies. Atala describes the socioeconomic status of her family as poor, and the researcher has had the impression of her living conditions as close to extreme poverty. Atala moved houses multiple times, and mainly lived in impoverished urban neighbourhoods with high crime rates. Before the pandemic, she supported her mother by making and selling charcoal and paper bags. Thanks to her maternal grandmother's support, and her mother borrowing money from multiple creditors, Atala was able to reach S.4, sitting her O‐Level exams shortly before the pandemic. Because of her many responsibilities at home, from childcare to household chores and helping her mother in income‐generating activities, Atala performed very poorly in her S.4 examinations, shattering her hopes of enrolling in a government‐sponsored nursing course. Shortly before the first lockdown in March 2020, Atala and her family had just moved to Bombo, a small town 30 km north of Kampala, where they found a cheaper and better house. However, the move required Atala's mother to commute for significant periods to Kampala for her tea selling business, leaving Atala at home with caretaking responsibilities of her younger siblings. When Atala had just enrolled in a government‐sponsored vocational training programme in tailoring, the first lockdown and closure of all schools was imposed, which hit the family particularly hard.

My mother was at home not working, it was tough for us to the point that my mother sent us to her mother's place where we could at least get something to eat. I felt so bad because we left our mother behind with no hope that food will come in from here or there. She said for her being an adult she could bare starving for even 2 days but my little siblings could not, they needed to eat no matter what.

After not having eaten for 2 days, several creditors asking for their money, and all public transport on hold because of the lockdown, Atala's mother had no other choice but to walk from peri‐urban Bombo to the capital city Kampala (around 6 h by foot) to start selling tea again. When we asked Atala during the first lockdown how she and the family managed to cope with all these challenges, she referred to reading the bible and praying and further noted:

We are used to this kind of life of lacking. It is not our first time to suffer like this but we have lived such a life from way back then.

Not only was Atala's skills training programme interrupted, but also her paper bag and charcoal selling business collapsed because of the pandemic. When her vocational institute re‐opened in March 2021, the long distance to school due to her remote location brought along new challenges:

I was so much challenged by distance. While doing my tailoring course, I was staying in Kawempe [outskirts of Kampala] and training from Wandegeya [in the city centre]. I would walk from Wandegeya to taxi park then I get taxis going to Kawempe. Men used to disturb me a lot during that time.

Although Atala was eventually able to complete her training, she felt that, despite her certificate, she was not qualified to work as a tailor. Hardly any teaching took place due to the lockdown and the presidential elections held in January 2021. According to Atala, classes were rushed and staff already focused on taking in the next cohort. With her side‐businesses no longer operating, no means of making an income to support the family and save some money for fees to re‐enrol in S.4 to retake her exams, Atala felt hopeless and depressed. She expressed a strong feeling of guilt for not being able to support her hardworking mother.

Int.: In your current state, what kind of support do you think you need?

Atala: Haha, I do not even know the kind of help I need. I am so lost and empty, it's not that I am looking forward to anything maybe like resuming schooling when schools reopen; no I really do not know.

Int.: Oh sorry!

Atala: Maybe the support I need is taking me back to school because when I go back to school at least I can be hoping that after this period of time I will achieve this, I will have taken another step in life, but for now I am so stagnant and my mother does not have money to take me back to school.

Int.: If it is not about going back to school, what other support do you need?

Atala: Then a job to do.

Int.: Will that make you feel better about yourself?

Atala: Yes, the reason I want to study is to get a job in the end and support my mother by taking on some of the so many responsibilities she has. But my mother is carrying all these responsibilities by herself.

Int.: What have you done about this?

Atala: That is why I told you that I am so useless, I do not help my mother in any way but all I am doing is to eat food and wait for another day.

Her loss of hope and sense of being ‘useless’ signal how she has internalized the intensified inequities, feeling herself to blame for the multiple challenges they face. What will happen next in Atala's life remains uncertain.

Atala's already difficult socioeconomic circumstances (in the form of severe poverty, being raised by a single female parent, multiple responsibilities at home or low to no income), were further challenged by being female as it is more difficult for girls to find a reliable source of income. In addition, her remote location not only lessened her chances of making an income, but also put her at risk of being sexually harassed during long commutes. Overall, not having access to truly free secondary education makes it extremely challenging for Atala to re‐enrol in school, follow her dream of becoming a nurse and generate a reliable income for herself and her family. The only fee‐free training programme that was available to her, in‐between lockdowns, was so compromised by the pandemic that it did not provide her with the very skills needed to earn a living. At the same she neither had the financial means nor the clientele, due to her remote location, to start a business of her own. Her experience is a reminder that inequity is a process and not a single event, and that frequently it cannot be mitigated with just one intervention (in Atala's case free access to a vocational training programme). Her experience showcases why ‘one‐size‐fits‐all’ approaches in education are not benefitting everyone in the same way. For a young female already living in extreme impoverishment prior to the pandemic; economic insecurities combined with remote location and gendered norms and inequities created considerable distress during the pandemic. These intersections significantly lowered her opportunities for an education according to her aspirations.

### How the intersections of socioeconomic circumstances and location create inequities in distance learning

The GoU (Government of Uganda) has made efforts to support learning during school closures through TV, radio or newspapers, and through making available downloadable curricula.^9^ In some instances, schools also have offered learning content via mobile phones. However, distance learning interventions, in particular, those who require having access to a mobile phone or a computer have been critiqued for only aiming at and benefitting urban elites. Most of our study participants had no or limited access to the resources needed to engage with these materials and supports—depending on their socioeconomic circumstances, location and to an extent also gender. Nakintu, for instance, a young female from a remote area and humble family background told us:

It being that we are in a rural community, our radio signals are weak and again we don't have a TV set. So as for me I am only relying on notes from school.

The intersection of being female and living in a remote area disadvantaged girls in particular as they tended to have less access than boys to mobile phones, as discussed in the next section. Apart from the multiple challenges to access learning content via TV, radio, mobile phones or the Internet, almost all school‐going participants told us that they could also not afford to buy newspapers or pamphlets with exercises. A few participants were attempting to continue studying by revising their school books, but were frustrated by having no access to teachers or relatives able to help. Having a family member who was available and able to engage in continuous home‐schooling is a privilege none of our study participants had. Young people also complained about not finding the time for remote learning, as they had to help their caregivers with household chores or engage in income‐generating activities. Nakintu spoke of how difficult it was to concentrate on studies when tired from working in the garden or while doing other household chores:

Nakintu: I miss studying as well.

Int.: But you told me you are revising your notes from home?

Nakintu: It does not match up with one who is revising from school. At school, you do not have other chores to do but only revision yet at home you have chores to first do and by the time to get to revise your books when you are so exhausted. As a result, you may just doze off in your attempt to revise your books from home.

Int.: Are you still interested in schooling?

Nakintu: Of course, I very much want to go back to school and finish up my O level studies then I join A level.

Shortly before the second closure of schools, Nakintu managed to sit her S.4 exams. Nonetheless, the subsequent lockdown and renewed closure of schools made her decide not to continue with her secondary education due to the before mentioned problems. Her plan is now to enrol in a vocational training programme instead. Nakintu's experience exemplifies how the intersections of being poor, living in a rural area and her family's expectations of her as a female household member to help with chores, compromised her ability to engage in distance learning materials and later continue with her education in multiple ways.

These intersections highlight the need for new and different approaches to help young people (in particular females, like Nakintu), to continue with their learning. In future epidemics or pandemics, one approach could be to explore the feasibility and safety of engaging teachers to support locally organized, small learning groups for children and youth to engage in outdoors teaching and learning. This would in particular help young people from a poor socioeconomic background, in remote areas with no infrastructure and technology to access digital learning. Having a regular and more formal engagement with teachers during periods of lockdowns may also help to manage caregivers' expectations on how much time their children have for household chores, or any other income‐generating work. Regular and more formalized learning groups could potentially also save females from being mainly confined to their homes, and males from migrating to other areas to engage in precarious work—as we briefly discuss below.

### How the intersection of gender and socioeconomic circumstances deepened inequities during school closures

For many of the young people we interviewed, the most significant effect of the lockdown on their lives related to the loss of family income. No longer being able to go to school exposed female and male adolescents to different stresses to help their families make ends meet. Their accounts suggest how deeply rooted gender inequities, including unwanted pregnancies, early marriage, unrecognized domestic or other work, or pressure on young males to provide an income, may have further deepened during the pandemic.

Atala was unusual among girls, for engaging in multiple income‐generating activities prior to the first and second lockdown. In the end, all her businesses collapsed because of the pandemic. By contrast, several male participants were able to make an income during school closures to either contribute to costs of schooling later on or support their families. At times they described their work as precarious and piecemeal. Kato (male, aged 18) shared with us:

Int. Have you been doing anything that has been giving you some cash?

Kato: In the last month, they took me to Gayaza and I spent a whole month there and I was a potter, helping in construction and maybe working from home here, because they put off our iron sheets on the house but as you know, when you work for the home people, you more or less work for free. Looks like you have worked for the church.

Int. So they did not pay you?

Kato: They did not. I worked for about 12 days.

Kato was eventually paid but his experience of exploitative labour was quite common. Tom (male, aged 18) had to find work to help his family make ends meet, especially because his mother and little sister had fallen ill and needed to buy medicine during the first lockdown. In November 2020, he told us that he had worked on a farm over the summer months but soon escaped as he was not paid, often beaten up for mistakes or being slow, not given any rest and not enough food. Ultimately, he found paid work in brick‐making, which allowed him to save up enough money to pay for just one single school term, which was soon interrupted when the second closure of school was imposed in June 2021.

Paid labour was less common among girls, and female participants generally reported being more confined to their home than boys, with less access to phones. Only five of the 18 (in and out of school) girls interviewed owned a phone (compared with 8 of the 14 boys interviewed), showing a similar gender disparity as CoVAC's quantitative survey sample (64% boys and 39% girls of 2403 young people surveyed in 2018 owned a mobile phone). Consequently, girls interviewed during the first lockdown seemed less able to stay in touch with friends. Those who had moved from urban to peri‐urban or rural areas during the lockdown were especially affected, with young women, in particular, speaking about how they missed meeting up with friends. Juliet, for instance, shared how the closure of schools and the church meant that she was suddenly socially isolated:

Int. There is a time you told me that most of your time if you are not at school, you spend it at church, now schools are closed and churches too. How has not being able to go to church affected you?

Juliet: It has affected me in a way that I am lonely I no longer get to see my friends.

Int. Do you mean to say that you can no longer meet up with your friends like before?

Juliet: Yes

Int. Why so

Juliet: Because most of the time we would meet at church so right now I have no way of seeing any of them because some of them probably went back to their homes

Educational institutions (alongside churches) were often described as the only space which allows girls to meet with their friends, whereas boys tend to enjoy more freedoms and are allowed to move around in the community and socialize. Our interviews suggest that caregiver constraints on girls' freedom of movement might have been aggravated by perceived risks for their daughters, such as the fear of teenage pregnancies. Four of our 36 participants (2 female, 2 male) recounted that they or their girlfriends got pregnant during the lockdown, and many more spoke of pregnancies among their peers, often seen as deriving from girls in economically constrained circumstances during the lockdown seeking material help from young men. Our data accord with a study by the WHO (World Health Organization), confinement to their homes during the pandemic all over Africa also reduced young women's access to crucial healthcare services (especially during birth) and posed many limitations for females to either engage in or continue with income‐generating activities.^10^ Overall, school closures combined with financial pressures, stressful family situations and more free time added to the complex mix of reasons for getting pregnant; showcasing the importance of contextualizing gendered experiences by paying attention to intersecting factors that produce inequities.

## CONCLUSION

While previous literature has convincingly linked Covid‐19 to the many losses and harms children and youth faced in and outside their education (see for instance: Alam & Tiwari, 2021; Datzberger & Parkes, 2021, Edwards, 2021; Save the Children International, 2021; UNICEF, 2021), our intersectional lens contributes to this emerging body of work by examining the complex layering of gender, location and socioeconomic circumstances that contributed to varying and nuanced experiences of inequities among youth prompted by the prolonged closure of schools. This has helped us to understand how grievances young people experience are interlinked and why they feed into inequities in their lives. For example the immediate effects of the pandemic on young people's socioeconomic circumstances required, more than ever, that they would find ways to generate an income during periods of school closures, which yet posed different challenges on the basis of their gender or location. For our male participants, such as Tom or Kato, this meant migrating from a rural area to another region to find work (which turned out to be precarious and exploitative), whereas for females mostly confined to their homes, or in rural areas, their options to earn some money were extremely limited. Rural location also challenged young people's access to distance learning, with weak radio signals. This apparent and often internalized ‘double‐failure’ of not being able to complete their education, nor finding or being allowed to work (mainly affecting our female participants), or failing to earn enough due to an exploitative work environment (mainly affecting our male participants), is a heavy burden to carry for our study participants. For Atala, this led to feelings of self‐blame for not being able to finish her education or engage in lucrative work. In other instances, as reported by Cathy, Kato or Nyanja, the many challenges concomitant with the closure of schools, dampened their future aspirations.

Our analysis further showed that the intersections of gender, socioeconomic circumstances and location, have a bearing on the effectiveness of interventions such as access to distance learning or free educational training programmes. Atala's case illustrates that free access to a vocational programme does not necessarily give all girls the same opportunities, especially if they live in remote areas or lack social and economic support. Already prior to the pandemic it would have been difficult for Atala to generate a sustainable income as a tailor, with her many responsibilities at home, living in a rural community with no financial means, tools or opportunities to start her own business. Covid‐19 rules and regulations clearly worsened her situation in that the training programme did not even sufficiently teach her the skills she was supposed to learn. Generally, the evidence is weak on whether formal and informal skills trainings empower in particular girls to find a sustainable source of income to support themselves (e.g. Annan et al., 2006, 2008). These programmes were also often disrupted in the extended lockdowns.

Moreover, our study participants' accounts on distance learning suggest that the implications for future interventions are many, if the same mistakes are to be avoided in new epidemics or pandemics. One way to help disadvantaged youth, such as Nakintu, to continue with their learning, could be to establish partnerships with local agencies and community‐based organizations to facilitate radio, TV or Internet‐based learning spaces. Another option could be to explore the feasibility and safety on in‐person gatherings, or even to consider the potential for regular outdoors teaching and learning.

To conclude, the interrelationship of economic circumstances, gender norms and relations as well as location and movement allows for new insights into the ramifications of the pandemic. It further helps to move away from binary, or simplistic, assumptions about the effects of school closures on the basis of gender, to better understand how gendered experiences are also linked to existing socioeconomic structures, systems, location and power. An intersectional approach is thus useful to recognize the differentiated nature of children and youth's vulnerability in extremely difficult situations. By explicitly paying attention to nuances and individual lived experiences, it provides a deeper understanding of how inequities and injustices arise and are further intensified by specific events. Strategies and interventions to overcome pre‐ and post‐pandemic social injustices can learn a lot from an intersectional lens on the basis of young people's accounts, their specific challenges, unique circumstances and everyday realities. Equally important, analysing inequities from an intersectional perspective reveals why some strategies are not addressing the root causes of pre‐existing and persisting grievances that affect and disadvantage adolescents.

## FUNDING INFORMATION

Medical Research Council (Grant number: MR/R002827/1). See https://gtr.ukri.org/projects?ref=MR%2FR002827%2F1.

## CONFLICT OF INTEREST

N/A.

## ETHICS STATEMENT

CoVAC (Context of Violence in Adolescents Cohort Study) was reviewed and approved by the following ethics committees: London School of Hygiene and Tropical Medicine (LSHTM), University College London—Institute of Education (UCL‐IoE), MRC/UVRI and LSHTM (Medical Research Council/Uganda Virus Research Institute and London School of Hygiene and Tropical Medicine Research Unit) and the Ugandan National Council of Science and Technology (UNCST).

## CONSENT STATEMENT

All young people who participated in the study were asked to provide consent and consented to take part. Most participants were between 15 and 16 years at fieldwork 1, and will be 19–20 by the end of the study. As this is a low‐literacy setting, we notified caregivers that the research is taking place by at least one of the following methods: providing a written notice to the participant to take home, via phone contact by the school headteachers or local chairperson, or by an information meeting. Caregivers can opt their children out of participation. All three methods are used to ensure maximum reach and to include caregivers with varying levels of literacy and engagement with schools.

For those living with the caregivers, who request to meet at home, upon arrival at the home we notified caregivers by providing (and reading aloud) a parental notification sheet.

Participants themselves were then asked to provide consent. For situations where adolescents are found living with marital/intimate partners, where they have their own children or are pregnant, are in child‐headed households, or are found in employment, including domestic work, we treated these adolescents as emancipated minors in accordance with the Uganda National Council for Science and Technology's classification. The Uganda National Council for Science and Technology defines emancipated minors as children below the age 17 years and are pregnant, married or have a child or cater for their own livelihood (http://research.ihsu.ac.ug/files/REC‐Documents/National‐Guidelines‐for‐Research‐Involving‐Human‐Participants.pdf). These out of school participants themselves will be asked to provide consent.

## PERMISSION TO REPRODUCE MATERIAL FROM OTHER SOURCES

Not applicable.

## Supporting information


Appendix S1

## Data Availability

Our data should be suitable for sharing in anonymized form. The study investigators support the principle of open access. Anonymized data will be offered to the UK Data Service, and will also be made available through the LSHTM research data repository. These services publish descriptive metadata in formats suitable for discovery (such as Dublin Core) and are indexed by search engines. Each data record will be assigned a Digital Object Identifier (DOI) for use in Data Access Statements and other publication citations. Data can also be made available through the MRC gateway or other initiatives, as appropriate. Our data sharing policy will be made available on all of these sources. A controlled access method will be necessary. To meet participant ethical obligations, data analyses must relate to improving health and educational outcomes in Luwero District (where the data are collected). Potential users of the data will be asked to provide information on their aims and proposed analyses of the data. The project team, including a representative of the LSHTM and Raising Voices, will evaluate the request and make a decision on access. The study team will retain exclusive use of the data until 2033. Provision for data linkage and longitudinal analysis has been included in the participant consent form. Users will be asked to sign an agreement that guarantees participant confidentiality and fulfils the objectives outlined in Governance of Access.

## Endnotes

^1^
We reviewed literature using the search words: ‘Covid‐19’ and ‘education’ and ‘Africa’ and in a second search also included ‘equity’ in Scopus and Google Scholar.

^2^
See https://en.unesco.org/covid19/educationresponse#durationschoolclosures, accessed 20 April 2022.

^3^

https://raisingvoices.org/, accessed 25 August 2021.

^4^
Percentage retrieved from: http://hdr.undp.org/en/countries/profiles/UGA, accessed 26 November 2021.

^5^
Enrolment in secondary school regardless of age and grade expressed as a percentage of the official secondary school going population of 13–18 years.

^6^
Senior refers to the specific grade in the secondary school system of Uganda. A secondary school is equivalent to a high school, and follows after 7 years of primary school.

^7^
See https://www.monitor.co.ug/uganda/news/national/school‐reopening‐master‐plan‐leaks‐‐3284472, accessed 2 December 2021.

^8^
See https://en.unesco.org/covid19/educationresponse#durationschoolclosures, accessed 19 April 2022.

^9^
See http://www.education.go.ug/covid‐19‐sector‐response/, accessed 26 November 2021.

^10^
See https://www.afro.who.int/news/who‐concerned‐over‐covid‐19‐impact‐women‐girls‐africa, accessed 02 December 2021.
