# Supplementary material for: Intensified inequities: Young people's experiences of Covid‐19 and school closures in Uganda
Source: Child Soc. 2022 Sep 5:10.1111/chso.12627. Online ahead of print. doi: 10.1111/chso.12627 (PMC9538397; doi:10.1111/chso.12627)
Supplement: Supplementary file 1 — Appendix S1 [file CHSO-9999-0-s001.docx]

Annex 1 and Annex 2

| **Annex 1:**  **List of CoVAC qualitative core participants either in school or planning to re-enroll after first lockdown** | | | | |
| --- | --- | --- | --- | --- |
| **Pseudonym** | **Gender** | **Age**  **2021** | **Urban /**  **Rural** | **Grade prior to 2^nd^ lockdown (June, 7^th^ 2021)**  **S=Senior*** |
| Mugera | Male | 18 | Urban | completed S4  vocational training |
| James | Male | 19 | Urban | completed S4  vocational training |
| Kalungi | Male | 19 | Urban | S5 |
| Nyanja | Male | 19 | Rural | S4 |
| Dan | Male | 19 | Rural | S6 |
| Kato | Male | 18 | Urban | S4 |
| Tom | Male | 18 | Rural | S3 |
| Kayondo | Male | 17 | Urban | S4 |
| Chekurut | Male | 19 | Rural | S5 |
| Peter | Male | 20 | Rural | vocational training |
| Atala | Female | 19 | Rural | Completed S4  vocational training |
| Nkola | Female | 18 | Urban | S3 |
| Apio | Female | 17 | Urban | S5 |
| Otim | Female | 20 | Rural | S5 |
| Juliet | Female | 18 | Rural | S4 |
| Mukake | Female | 18 | Rural | S4 |
| Nakintu | Female | 17 | Rural | S4 |
| Nakafeero | Female | 18 | Rural | S3 |
| Jane | Female | 18 | Rural | S4 |
| Cathy | Female | 18 | Urban | S5 |
| Ruth | Female | 18 | Urban | S4 |
| Nankoma | Female | 19 | Rural | S5 |
| *Senior refers to the specific grade in the secondary school system of Uganda. A secondary school is equivalent to a high school, and follows after six years of primary school. | | | | |

| **Annex 2: Overview of past and ongoing CoVAC (qualitative) data collection 2018-2021** | |
| --- | --- |
| **Fieldwork 1**  October – December 2018 | - Face to Face interviews with 36 core participants (2-3 hours) - Community walks with 36 core participants (1-2 hours, followed by 1-2 hour interviews) - 33 caregiver interviews (1-2 hours) - 6 FGDs (1-3 hours) - All data translated, transcribed and coded in Nvivo - Biographical summaries and fieldnotes were created |
| **Informal stay in touch calls**  May – June 2019 | - Short phone conversations (10-20 minutes) - Not transcribed or coded but information from call was added to biographical summary |
| **Fieldwork 2**  October – December 2019 | - Face to face interviews with 35 out of 36 core participants (2-3 hours) - 9 teacher interviews (1-2 hours) - 8 stakeholder interviews (1-2 hours) - 4 FGDs (1-3 hours) - All data translated, transcribed and coded in Nvivo - Biographical summaries and fieldnotes were updated |
| **Formal stay in touch calls**  May – June 2020 | - Longer phone conversations (30-45 minutes) with 34 out of 36 participants - All data translated, transcribed and coded in Nvivo - Biographical summaries and fieldnotes were updated |
| **Fieldwork 3**  October – December 2020 | - Face to face or phone interviews with 35 core participants (1-2 hours) - 9 peer interviews (1-2 hours) - 10 teacher interviews (1-2 hours) - All data translated, transcribed and coded in Nvivo - Biographical summaries and fieldnotes were updated |
| **Formal stay in touch calls**  May – August 2021 | - Longer phone conversations with 35 out of 36 participants (30-45 minutes) - All data translated, transcribed and coded in Nvivo - Biographical summaries and fieldnotes were updated |
